# Supplementary material for: Are carbon emissions trading and green financial instruments synergistic? -Comprehensive quantitative research based on content analysis
Source: PLoS One. 2024 Mar 7;19(3):e0298601. doi: 10.1371/journal.pone.0298601 (PMC10919601; doi:10.1371/journal.pone.0298601)
Supplement: S1 Data — (ZIP) [file pone.0298601.s001.zip › Data/地级市Z_F工作报告.zip!/║■▒▒/╗╞╩»╩╨/└┤╫╘╣1⁄2╓┌║┼í╛┬φ┐╦╩2╛▌═°í┐_2014.docx]

各位代表：

现在，我代表市人民政府向大会报告工作，请予审议，并请市政协各位委员和其他列席人员提出意见。

一、2013年工作回顾和2014年工作总体要求

2013年是全面贯彻落实党的十八大精神的第一年。一年来，在市委的正确领导下，在市人大、市政协的监督支持下，我们紧紧依靠全市人民，坚持生态立市、产业强市，对接武汉、比学九江，竞进提质、速效兼取，经济社会保持平稳较快发展，实现了建设鄂东特大城市的良好开局。初步预计，全市完成生产总值1144亿元，比上年增长10.5%；规模以上工业增加值572亿元，增长11%；全社会固定资产投资960亿元，增长28.7%；社会消费品零售总额450亿元，增长12.5%；外商直接投资4.9亿美元，增长16.7%；外贸出口总额12.1亿美元，增长10%；地方公共财政预算收入78.36亿元，增长19.38%；城镇居民人均可支配收入21553元，增长11%；农民人均纯收入8374元，增长12%；城镇登记失业率控制在2.18以内；人口自然增长率7.83‰。

(一)产业转型取得新成效。坚持改造提升、集群发展，大力实施工业“两计划两工程”1。实施亿元以上产业转型项目200多个，淘汰落后产能1.5万吨，5个产业列入省级成长型产业集群；新增高新技术企业23家，高新技术产业增加值178亿元，增长14，占GDP比重达到15.5%，居全省第三位；优化对重点企业的服务，筹集资金1.5亿元，通过财政贴息等方式支持企业度难关、保增长，设立中小微企业发展调度资金5000万元，全市担保机构担保总额80亿元，增长23.5%，新增规模以上企业 129 家，12家企业入选全省百强，数量居全省第二。加快推进企业上市，芳通药业等4家企业在武汉股权托管交易中心实现挂牌交易。

实施质量兴市、品牌强市战略，新冶钢荣获全省第四届长江质量奖2，新增中国驰名商标4件。积极实施农业倍增计划，着力发展现代农业，新增土地流转面积2.3万亩，农民专业合作社突破千家达到1042家，新增省级农业产业化龙头企业5家，战胜了历史罕见的旱情和高温热害，粮食生产实现十连增。加大对服务业发展的政策支持力度，新增限额以上商贸企业140家、限额以上服务业企业120家，第三产业增加值占GDP比重达到30.8% ，比上年提高0.8个百分点。口岸核心能力建设通过国家验收。全年金融性投入1266亿元，增长22%。区域经济加快发展，大冶市在全国百强排名中实现进位，阳新县综合实力在全省排名进一步提升，各城区呈现竞相发展良好态势。

(二)招商引资和项目建设实现新突破。创新招商引资方式，组建10个招商分局、54支招商小分队，围绕10大主导产业，强力推进产业链招商，招商引资规模和质量显著提升，全市新签重点合同项目120个，实际到资630亿元，增长96.9%，省外资金实际到资318.6亿元，增长1.2倍，成功引进正威电子、大连万达、华纳国际铜箔等一批重大项目。深入开展重大项目建设年活动，实施亿元以上项目420个，增长65%，新开工重点项目270个，增长54%。大力推进园区建设，全市9个省级以上工业园区聚集各类企业5700家，规模以上工业企业单位数占全市的80%以上；黄石(B型)保税物流中心3成功获批并开工建设，棋盘洲港区一期、新兴际华现代钢铁物流园等重点项目加快推进。积极向上争取政策资金，黄石列入全国老工业基地调整改造规划、资源城市可持续发展规划，争取国家、省科技计划项目74项，获得省级以上重大成果120项。

(三)城镇化建设迈出新步伐。城市总体规划修编和大冶湖生态新区概念性总体规划编制工作全面完成，数字黄石基础平台4和城市规划一张图系统5基本建成，黄石区域规划基本实现全覆盖。综合交通运输体系日趋完善，黄咸高速大冶段建成通车，武黄城际铁路全线建成，月亮山隧道、316国道黄石段、黄石(铁山)至武汉(光谷)一级公路等重点项目开工建设，长乐大道一期、河西大道主体工程竣工，社区环境综合整治和背街小巷、农贸市场、环卫设施改造等民生工程进展顺利。加强农村基础设施建设，建成一、二级公路路基110公里、路面74公里和农村公路357公里；完成各类大小水利工程6800余处，新增、改善、恢复灌溉面积14.8万亩，新建农村集中供水工程24处，解决10万农村居民、2万农村学校师生饮水不安全问题。农村集体土地两权登记发证工作加快推进。扎实开展新三万活动，投入资金2亿多元，大力推进新农村建设，乡村环境明显改善。

(四)生态文明建设取得新进展。启动编制生态立市产业强市总体规划。实施生态环境修复治理工程，治理矿山面积8718亩，全市6家企业成功创建国家级绿色矿山，新增造林面积5.95万亩，改造和新增绿地面积64公顷，森林覆盖率比上年提高0.6个百分点。成功举办生态黄石园林花卉展。加强土地节约集约利用工作，工矿废弃地一期复垦项目顺利通过验收，荣获全国国土资源节约集约模范市。扎实推进工程减排，实施新兴管业高炉系统优化、西塞电厂3号机组脱硝、华新水泥(阳新)脱硝等重点项目，强化污染源在线监控建设管理，取缔关闭五小企业6278家。大力发展循环经济，支持华新水泥、大冶铁矿等企业加强资源综合利用，大冶有色城市矿产7示范基地项目加快实施，黄石列为国家循环经济示范创建城市。水污染综合治理利用亚行贷款项目稳步推进，环磁湖养猪场全部关停，河西污水处理厂加快建设，新建污水管网40公里，磁湖、青山湖、大冶湖水质趋于好转。万元生产总值综合能耗及主要污染物减排完成省定目标。创新环境执法体制机制，在全省率先成立环保警察支队，严厉查处环境违法行为。

(五)发展环境实现新优化。组建黄石市商务委员会、黄石市招商局，形成大商务、大招商工作格局。坚持简政强园、托管扩园，对开发区、下陆区、黄石港区和东方山景区管理体制进行调整，将阳新两镇一区、西塞山章山片区委托开发区管理，拓展了开发区发展空间。推进简政放权，将18个政府部门45项职权全部下放区级政府行使，出台支持开发区、阳新县和西塞山区发展的政策，促进区域经济快速发展。大力开展春雷行动，全市58个职能部门作出公开承诺，取消和减免涉企收费(基金)122项，在全省率先实现涉企行政审批零收费。对全市53家审批职能部门622个行政审批(服务)事项进行全面精减，精简幅度达到50%。金融生态环境进一步优化，黄石连续9年被评为全省金融信用市。发展环境的明显好转，激发了全社会投资热情，全市新登记各类市场主体5万户，增长87.6%。

(六)民生福祉得到新改善。全市各级财政用于民生支出达到116亿元，增长12.6%。各项社会事业全面发展，学前教育三年行动计划8加快实施，义务教育标准化学校建设扎实推进，高中阶段毛入学率达到93.5%，职业教育、高等教育进一步发展。公共卫生服务能力得到提升，公立医院改革试点工作取得新进展，新农合参合率稳定在95%以上。公共文化设施建设明显加强，黄石成为全省唯一的国家首批公共文化服务体系示范区城市，华新水泥厂旧址、大路铺遗址列为第七批全国重点文物保护单位。群众体育活动蓬勃开展，成功举办黄石市第九届运动会。深入推进全国创业先进城市建设，扶持成功创业8379人，带动就业2.9万人。全市五项社会保险参保总人数达247万人(次)，征缴社会保险费35.2亿元，城乡居民社会养老保险参保率达到99.97%。顺利完成企业退休人员养老金调标任务，市本级人均养老金达到1629元/月。认真实施医保新政策，全市14703名企业退休人员免费享受医保待遇。社会救助工作进一步加强，建立慈爱方舟大病医疗救助基金9，市居家养老呼叫服务中心开通，一批社区日间照料中心和社区养老服务中心建成并投入使用，黄石成为全省唯一的全国社区治理和服务创新试验区城市。保障性安居工程建设取得新进展，全市开工各类保障房8542套，竣工各类保障房9251套，新增租赁补贴户779户，黄石保障性住房建设经验在全国推广。切实加强和创新社会管理，积极排查化解矛盾纠纷，持续开展严打整治，全面推进动态化、立体化社会治安防控体系建设，社会治安状况明显好转。公共突发事件应急体系不断完善。安全生产形势持续稳定好转。食品药品安全监管力度进一步加大。残疾人保障和服务体系建设顺利通过省级验收。扎实开展第三次全国经济普查工作。人口和计划生育、审计、广播电视电影、对台、保密、档案、妇女儿童、青年、老龄、援藏、援疆、援利川、外事、侨务、旅游、民族、宗教、国家安全、编制、科普、防震减灾、民防、水文、气象、史志等工作都取得新的成绩。市政府承诺的十件实事22个子项目中除青山湖2、3、4号湖清淤及处置工程没有完成外，其他各项实事均已完成。

过去的一年，我们高度重视政府自身建设，着力健全政府议事和决策规则，自觉接受市人大及其常委会的法律监督、工作监督和政协的民主监督，主动听取民主党派、工商联和社会各界意见和建议，人大代表议案、建议和政协提案办复率达到100%。精神文明和民主法制建设深入推进，反腐倡廉力度加大。国防动员和后备力量建设进一步加强，民兵建设试点经验在全省推广，双拥优抚、军民共建工作扎实推进，军政军民关系更加密切。

奋斗历经艰辛，成绩来之不易。过去的一年，黄石经济社会发展保持良好势头，这是省委、省政府和市委坚强领导的结果，是市人大、市政协有力监督支持的结果，是全市广大干部群众共同奋斗的结果。在此，我代表市人民政府，向全市人民，向给予政府工作大力支持和有效监督的人大代表、政协委员和民主党派、工商联、无党派人士，各人民团体，向驻黄解放军、武警官兵，向所有关心支持黄石建设和发展的海内外人士，向在东楚大地投资的创业者和建设者，表示衷心的感谢!

在看到成绩的同时我们也清醒地认识到，前进道路上还有许多困难和问题，一些深层次矛盾还未从根本上解决。受宏观经济环境的影响，经济下行压力较大，这对黄石转型跨越、争先进位是个严峻考验；体制机制性矛盾仍然突出，经济结构调整任务艰巨，生态环境保护压力加大，深化改革开放和加快转变经济发展方式更为迫切；城乡居民收入水平不高，部分群众生活还比较困难，基本公共服务体系尚不健全，民生改善与人民群众的期盼还有差距；个别地方和部门官僚主义、形式主义甚至贪污腐败现象依然不同程度存在，行政效率和公务员素质有待提高。对这些问题，我们将采取有效措施认真加以解决。

2014年，是全面贯彻落实党的十八届三中全会精神、全面深化改革的第一年，也是全面完成十二五规划的关键之年，做好今年的工作，意义重大。今年政府工作的总体要求是：认真贯彻落实党的十八大、十八届三中全会和中央、全省经济工作会议精神，牢牢把握稳中求进、改革创新的工作总基调和竞进提质、升级增效的工作总要求，坚持生态立市产业强市，以全面深化改革统揽全局，以转变经济发展方式为主线，统筹城乡发展，扩大对外开放，着力改善民生，加快建设鄂东特大城市步伐，努力实现经济社会平稳较快发展。

综合考虑当前的宏观发展环境和黄石的现实基础，2014年全市经济社会发展的主要预期目标是：地区生产总值增长10%；规模以上工业增加值增长12%；全社会固定资产投资增长25%以上；居民消费价格涨幅控制在4%左右；社会消费品零售总额增长14%；地方公共财政预算收入增长12%以上；外商直接投资实际到资增长12%，外贸出口总额增长13%；城镇居民人均可支配收入增长11%以上，农民人均纯收入增长12%以上；城镇登记失业率控制在4.6%以内；人口自然增长率控制在8.5‰以内；确保完成省下达的节能减排任务。

二、全面落实生态立市产业强市战略举措

(一)加快推进产业转型升级

着力推进新型工业化。以信息化与工业化深度融合为方向，深入实施两计划两工程，着力抓好新冶钢特殊合金棒材技改、东钢搬迁、新兴管业环保升级整体搬迁等改造升级工程，加快推进正威电子信息、黄石生物环保等产业园建设，大力实施沪士电子、华纳国际铜箔、斯堪维亚机床、西塞山电厂二期等重大项目，促进产业链延伸和集群发展，逐步走出经济发展对采矿经济的路径依赖。坚持一企一策，进一步完善重点企业直通车服务机制，突出抓好小微企业协调服务，力争今年新增规模以上企业130家以上。认真贯彻国家、省淘汰落后产能政策，确保完成省定任务。积极申报国家工业绿色低碳转型发展示范市，着力推进绿色低碳发展。积极申报国家住宅产业化综合试点城市，加快具有黄石特色的住宅产业化步伐。

大力实施创新驱动战略。加快推进高新技术产业化，支持挤出模具、汽车零部件、循环经济等产业进入省级高新技术产业化基地行列，实施科技型中小企业成长计划，力争全年新增高新技术企业15家以上，高新技术产业增加值占GDP比重提高0.5个百分点以上。加快组建黄石智能输送装备、特殊钢、涂镀板、畜禽等产业技术创新战略联盟，推进中科院湖北产业技术创新与育成中心黄石分中心建设。支持企业加强创新能力建设，支持企业申报国家级、省级企业技术中心和工程实验室，支持劲牌、鑫东等企业申报承担国家和省级重大科技计划项目。加强创新创业人才队伍建设，培养和引进高层次科技创新领军人才、学术技术带头人，引导科技人才向企业聚集。积极开展国家创新型试点城市创建。

突破性发展现代服务业。改造提升传统商贸流通业，加快推进原黄石实验高中片商贸项目的引进改造、万达广场、一轴摩尔城等项目建设，建成武商摩尔城，建设一批社区便利店、连锁店和农贸市场。以现代物流业、金融服务和信息服务为重点，支持蓝溪国际、鑫大地等物流企业扩大规模，加快推进阳新荻田物流产业园、棋盘洲粮食物流园等项目建设，引进国内外知名电商企业来黄建立物流仓储中心、区域结算中心，大力发展一体配送、电子商务、金融租赁、总部经济等新型业态。推进全国中小商贸流通企业服务体系建设试点，全面落实早餐工程、家政服务等方面的支持政策，做大做强服务业市场主体。大力发展文化旅游业，支持铁山区打造世界铁城，加快以东方文化园为核心的东方山景区开发建设，成功创建国家4A级景区；大力推进阳新仙岛湖蓝海一号、铁山喻家垅旅游文化产业园、东楚传媒产业园等项目建设。坚持对接大武汉、发展乡村游思路，全力开发沿长江、沿磁湖、沿大冶湖、沿城际线和沿高速路的乡村旅游示范点、农家乐和生态景区。积极发展养老和健康服务业。

(二)加快推进生态城市建设

着力完善城市生态功能。以创建全国生态文明先行示范区为抓手，按照方案化、项目化、工程化的要求，统筹推进国家生态市、全国文明城市、国家卫生城市、国家环保模范城市、国家森林城市等各项创建工作。积极申办湖北省首届园林(园艺)博览会，举办黄石首届园林(园艺)博览会。突出抓好自然生态系统和景观绿化建设，推进江边、湖边、铁路边、干道边、城镇边的绿化洁化美化，建设五边三化生态治理示范区，重点实施磁湖湿地公园、磁湖南岸滨水景观、老下陆生态新城、城市森林公园、团城山公园扩园等项目建设，完成大泉路、广州路、106国道、河西大道、矿冶大道二期等道路景观绿化工程。

加大生态环境治理保护力度。严格落实环境保护责任制，继续执行环境影响评价和三同时管理制度。制定实施治山、治水、治土、治气行动计划。继续实施矿山地质环境治理工程，按照有序、有限、有期的原则，关停沿江、环湖等重点区域80%以上矿山。大力实施内源削减、水体修复、生态调水等重点工程，加强以磁湖、大冶湖、网湖、保安湖和富河为重点的水环境保护与生态修复。严格执行危险废物经营许可证制度和联单管理制度，加大对重点污染源及危化品环境安全隐患排查力度，全面推进重金属污染防治。深入开展农村面源污染防治。制定雾霾综合治理措施，加强餐饮油烟污染、垃圾无序焚烧、农村秸秆焚烧治理，全面推进城区禁鞭，促进空气质量有效改善。扎实抓好节能减排，深入开展全市五小企业和工业企业重点污染源整治专项行动，关闭五小企业348家，整治工业企业重点污染源438家。加快实施燃煤电厂、水泥厂脱硝工程，中心城区全面实施65%低能耗建筑节能标准和绿色建筑评价体系，统筹推进机动车、建筑扬尘等污染物防治。积极创建国家循环经济示范市、全国新能源示范城市，加快推进大冶有色城市矿山示范基地等循环经济项目，形成循环经济产业链。

(三)加快推进新型城镇化

优化城镇布局和形态。强化以人为本、彰显特色的理念，按照集约发展、布局协调、结构合理的要求，启动编制新型城镇化总体规划，编制完成一批重点城镇规划。认真抓好省、市四化同步10示范乡镇试点建设，支持县域中心镇加快发展，提高城镇建设水平，努力打造产业支撑型、文化旅游型、生活宜居型等不同特色、不同风格的个性化城镇，加快建成一批综合实力突出、布局合理、规模适度、功能健全的中心镇或特色镇。着力推进100个美丽乡村建设，注意保留村庄原始风貌，慎砍树、不填湖、少拆房，尽可能在原有村庄形态上改善居民生活条件，让居民记得住乡愁。研究制定推进农业转移人口市民化的政策措施，进一步深化户籍制度改革，全面放开建制镇和小城市落户限制，创造公平的就业、就学、就医和居住环境，解除农民进城后顾之忧。

大力推进旧城改造。高起点编制城区旧城改造规划。抢抓全国推动棚户区改造政策机遇，推进磁湖北岸、财校片、市建村片、人民街等项目改造升级，启动实施十三排、王家湾等棚户区和胡家湾片区3000亩改造工程，打造磁湖商务区。以完善市政道路、管网、供排水等基础设施为重点，加大新、老下陆片区城市污水处理管网建设力度，改造冶钢泵站和河口、西塞、老下陆、东钢等4座排洪港，全年疏通排水管网60公里，确保城市排水畅通。推进城区背街小巷刷黑改造，维修改造城市道路、人行道2万平方米。认真做好三环锻压、新兴管业等合规企业退城入园扩能工作。

全面推进大冶湖生态新区建设。编制新区各层次专项规划，确保规划管理全覆盖。加快推进月亮山、黄思湾隧道和大冶湖南北滨湖大道等重点工程建设，稳步推进新区基础设施、公共服务设施和2平方公里核心区建设，拉开城市骨架、完善新区功能。全面治理环湖区域矿山开采和污水直排、违法填湖、围栏养殖等行为，实施退田还湖工程，推进环湖生态、绿色通道保护与建设。

着力推进城乡交通设施建设。完善中心城区路网系统，启动沿江大道工程建设，加快东方大道、发展大道、月亮山路灯道路建设改造。调整优化公交线路布局，积极推进社会停车场建设，新增停车场泊位500个，缓解中心城区停车难。完成全社会交通投资70亿元，开工建设棋盘洲长江公路大桥连接线、武九客运专线大冶至阳新段及沿江大道城区段，基本建成新港进港铁路。加快推进棋盘洲港区二期、团城山客运枢纽站等项目建设，做好河西大道综合改造工程收尾工作，完成黄富公路改造升级，建成106国道一级公路阳新至大冶段、阳新至武汉一级公路阳新段，力争开工建设黄石至阳新快速路，改造农村公路150公里。

切实加强城市管理。完善统一领导、分级负责，条块结合、以块为主的城市综合管理格局，加强数字化城市管理平台建设，提高精细化管理水平。实施城市管理疏导工程，制定便民市场布点规划，合理设置农贸市场、小商品市场。开展户外广告、占道经营等专项整治行动，提高城区道路通畅水平，确保市容环境整洁有序。建立农村环境卫生责任区制度和政府监管机制，完善城市垃圾收运体系，整体改善城乡环境卫生。

(四)扎实做好农业农村工作

稳定发展农业生产。扩大双季稻种植面积，减少弃耕抛荒，提高粮食综合生产能力。大力开展高标准农田、农业信息化和防灾减灾救灾体系建设，夯实农业发展基础。落实和完善各项强农惠农政策，积极探索惠农补贴资金有效整合的方式和途径。加快推进农业标准化生产，加强农产品质量安全监管，确保农产品质量安全。健全完善基层农技推广服务体系，提高主要农作物机械化水平。

加快发展现代农业。坚持以工业化、生态化、产业化理念发展现代农业。突出抓好大冶农产品加工和阳新现代农业示范园建设，壮大畜禽、食品饮料加工和现代渔业规模，培育和发展一批农业品牌。推广生态农业循环模式，提高畜牧业规模化发展准入门槛，积极发展休闲观光农业，建成一批星级山水农庄、度假村和休闲基地。推进油茶、花卉苗木、林下经济等涉林产业发展。支持工商资本下乡、能人返乡创业，加快土地向种植大户、专业合作社和龙头企业等流转集中，培育农业新的市场主体。大力发展县域经济，支持大冶市保百强、再进位，支持阳新县奋进全省县域经济二十强。

深入推进新农村建设。扎实开展万名干部进万村惠万民活动。开工建设水库除险加固、塘堰整治等各类水利工程2300处，着力改善农村生产生活条件。加快城镇垃圾污水处理设施建设，规划建设乡镇垃圾转运系统，支持大冶市推进村镇垃圾收运系统建设、阳新县加强偏远城镇垃圾简易处理设施建设，建成陈贵镇、灵乡镇、还地桥镇污水处理厂，启动富池污水处理厂建设。加快推进幕阜山片区扶贫开发。

三、全面深化改革开放

(一)强力推进重点领域改革

深化行政管理体制改革。全面清理、分批取消和下放投资项目审批、生产经营活动和资质资格许可等事项，试行委托社会组织代行，严格控制新增审批项目，推进工商登记制度改革，加快建立统一规范的公共资源交易市场，充分发挥市场在资源配置中的决定性作用。坚持重心下移、理顺关系，进一步转变政府职能，重点抓好西塞山区简政放权改革试点，支持西塞山区争创全省同类第一城区。抓好经济发达镇行政管理体制改革试点工作，全面赋予试点镇县级管理权限。积极稳妥推进事业单位分类改革，探索推行公务员聘任制和事业单位雇员制，推动公办事业单位与主管部门理顺关系和去行政化。推进行业协会改革，提高行业协会的专业化和社会化水平。

深化财税体制改革。进一步调整完善市本级与城区、开发区的财政管理体制。深入推进部门预算、国库集中收付、政府采购、收支两条线、行政事业单位国有资产管理和公务卡制度改革，完善全过程预算绩效管理，全面推行三公经费公开，确保财政资金使用透明、规范、高效。积极落实结构性减税政策，着力推进营改增试点改革。探索建立全市范围的不动产统一登记和信息公开制度。

创新投融资机制。健全多层次资本市场体系，推动企业上市、股权交易和企业债券发行，确保2-3家企业挂牌、上市，抓好市城投公司三期和众邦公司债券发行，积极引导社会资本参与城市基础设施建设。加强金融要素市场建设，增强金融资源集聚能力。探索建立农村产权抵押融资机制，开展森林政策性保险。完善融资担保补偿机制，进一步扩充全市担保机构资本金，扶持中小微企业发展。支持小额贷款公司发展。依托主导产业建立产业发展基金，引导和支持产业转型升级。规范全市融资平台建设，控制政府性债务风险。

深化国有企业改革。大力发展混合所有制经济，基本完成美尔雅改制工作，继续推进东贝集团、工矿集团等企业深化改革，鼓励非公企业参与国企改革，完善企业法人治理结构和现代企业制度。加快解决国有企业改革改制遗留问题。加大国有资产监管力度，完善国有资本经营业绩考核评价体系，确保国有资产保值增值。

深化民生领域改革。全面推进以管办评分离为重点的教育综合改革。深入推进医药卫生体制改革。进一步培育和发展社会组织，健全政府向社会组织购买服务机制，推进基本公共服务均等化。继续推进全市文艺院团等国有经营文化单位转企改制，推动政府部门由办文化向管文化转变。探索建立资源有偿使用和生态补偿制度，加强环境保护和环境执法体系建设。加快推进质量兴市工作，争创全国质量强市示范城市。深化食品药品管理体制改革，加强食品药品检验检测服务平台建设，建立统一的市场监管体系。创新基层社会管理和公共服务体制机制，积极鼓励和支持社会组织参与社会建设，不断完善多元社会治理结构，争创全国社会管理创新综合试点城市。

(二)全面扩大对外开放

加快推进沿江开放开发。抢抓国家依托长江打造中国经济升级版支撑带的战略机遇，按照江城联动、江湖联动、江路联动、江园联动的思路，编制沿江开发总体规划和交通集疏运建设等专项规划，关闭沿江非法及不符合规划的码头。统筹推进沿江产业发展，支持新港(物流)工业园围绕建设亿吨大港，加大招商引资和承接产业转移力度，推进口岸配套设施和海关特殊监管区建设，确保黄石(B型)保税物流中心6月30日前封关运行。支持黄石港工业园发展高新技术产业、西塞山工业园申报省级高新区、阳新滨江工业园发展医药化工产业集群，打造沿江经济走廊。加快推进进出口企业孵化中心和口岸大通关建设，加大外向型企业培育力度，支持十五冶、华新、有色等企业开展境外承包工程、境外投资等业务，推动外经外贸加速增长。

强力推进产业链招商和项目建设。按照投入产出、科技含量、集约用地、节能减排、税收贡献、建设周期六度评价标准，围绕10大主导产业，制定产业链招商行动计划，建立全市统一招商重点项目库和信息评估机制，进一步提高项目质量。完善产业链招商运作机制，加强信息收集-信息评估-产业链和环保审查-项目落地全过程统筹，积极探索企业化招商、中介招商、代理招商和网络招商等方式，切实提高招商实效，力争全年新签合同重点项目130个，新开工重点招商项目120个，实际到位资金700亿元以上。继续实行项目建设专题推进机制，加快推进716个、总投资2237亿元导向性计划项目，确保正威电子信息产业园、光谷激光技术运用产业园等395个、总投资1177亿元的在建项目顺利实施，永康五金机电城、浙楚联盟零部件精加工等321个、总投资1060亿元的重大项目开工建设。

加大园区建设力度。以基础设施建设、招商引资和项目落地为重点，统筹推进各类园区开发建设。市开发区重点抓好金山大道东延、庆洪路南延、章畈路等路网及配套设施建设，力争完成七通一平1万亩以上，引进项目合同投资额450亿元以上。工矿废弃地试验区重点推进光谷工业园区路网建设，确保综合治理示范工程通过省级验收。支持大冶开发区和灵成工业园提档升级，发展先进制造业和高新技术产业。支持阳新开发区发展机械制造、轻工纺织等主导产业，确保入园企业80家以上。支持下陆工业园发展循环经济。引导和支持各乡镇因地制宜发展特色园区，进一步壮大镇域经济规模。

(三)加快与武汉对接。完成黄石(铁山)至武汉(光谷)一级公路建设，加快基础设施对接。依托武汉大企业，积极引进汽车零部件、船舶、机械制造等项目，将总部在武汉的大企业生产基地争取到黄石，加快产业对接。大力引进武汉商业巨头来黄石发展连锁店，引导和支持黄石工业产品、农副产品、手工艺品等开拓武汉市场，加快市场对接。积极利用武汉金融、人才、劳动力市场，深化同武汉高等院校合作，加快技术和人才对接。

四、切实保障和改善民生

(一)深入推进创业带就业。全面落实创业扶持政策，扩大创业小额贷款担保基金规模，抓好创业孵化基地等服务平台建设，支持全民创业和小微型企业发展，提升创业质量。完善公共就业服务体系，多渠道开发就业岗位，加强高校毕业生、农村转移劳动力、城镇就业困难人员等重点群体就业援助工作，最大限度增加就业容量。健全职业技能培训与服务企业用工联动机制，提高劳动者就业能力，促进更高质量就业。

(二)扎实做好社会保障工作。进一步扩大社会保险覆盖范围，突出抓好扩面征缴和老工伤趸缴工作。落实退休人员免缴医保费等城镇职工医疗保险政策，整合完善新农合制度。完善劳动关系三方协调机制，规范劳务派遣用工行为，扩大工资集体协商覆盖面，着力构建和谐劳动关系。加快推进养老服务社会化。加强社会救助工作规范化建设，努力做到按标施保、应保尽保。完善住房保障五个一制度体系11，坚持增加保障房源和统筹管理各类房屋两手抓，进一步扩大住房保障覆盖面。

(三)完善基本公共服务体系。实施新一轮学前教育三年行动计划。深入推进义务教育均衡发展，确保标准化学校建设全面通过省级评估验收，大冶市通过国家评估验收。调整优化高中阶段学校布局，促进普职协调发展。支持高校加快发展，支持湖北城市职业学校创建国家级示范学校。严格规范学校办学行为，重拳整治教育乱收费行为。加快推进鄂东南区域性医疗卫生服务中心建设。积极引导社会资本进入医疗服务薄弱领域，构建多元化办医格局。扎实做好重大疾病防治和卫生应急工作。大力推进文化惠民工程，新群艺馆建成开放，图书馆、博物馆、文化站、文化室等重点文化设施得到充分利用。加大矿冶工业遗产申报世界文化遗产力度，做好华新水泥厂旧址、铜绿山古铜矿遗址等申遗核心项目建设和保护管理工作。全面开展全民阅读等群众文化活动，支持民间艺术团体健康发展，进一步打响黄石文化品牌。推进农村一村一月一场电影工作，启动农村广播电视户户通工程。全面完成国乒基地重建工程，加快推进南湖游泳馆、黄金山奥体中心建设，高质量承办全国乒乓球锦标赛，积极申办湖北省第十五届运动会。认真落实人口计生基本国策，突出抓好降低出生人口性别比工作，稳定低生育水平。启动实施残疾人同步小康工程，整体提升残疾人生活质量。

(四)创新社会治理。全面推进社区治理和服务创新，完善社区网格化管理，增强城乡社区居民自治和服务功能。加强网络舆论引导，提高应对虚拟社会的管理水平。畅通群众诉求渠道，加快推进行政复议规范化建设，健全人民调解、行政调解、司法调解联动工作机制，积极调处化解各类矛盾纠纷，维护群众合法权益。加强安全生产基层基础工作，抓好重点行业领域安全生产专项整治，完善消防救援体系。深入开展六五普法。扎实推进平安黄石建设，进一步完善动态化、立体化社会治安防控体系，严厉打击各类违法犯罪活动，确保人民安居乐业、社会安定有序。

(五)认真办好十件实事。(1)建设10处城市森林公园；新增、改造绿地面积60公顷。(2)实施铁山铁金港新下陆东西港污水干管工程，对接大冶城西北污水干管。(3)实现就业创业培训1.5万人，帮扶成功创业6000人，带动就业1.8万人；城镇新增就业4.5万人。(4)开工建设各类保障性安居工程13021套(户)，基本建成14030套(户)。(5)对100户贫困残疾人家庭实施无障碍改造；对200名0-6岁贫困残疾儿童提供抢救性康复救助；为贫困精神残疾人发放1000张服药卡；为贫困残疾人免费发放1000件辅助器具和为1000名残疾人提供托养服务；为残疾人提供职业技能和就业培训3000人次。(6)解决8万农村人口饮水安全问题；完成28个重点贫困村、老区村和插花村的脱贫解困工作，减少贫困人口1.1万人。(7)为5万名城乡贫困适龄妇女免费进行宫颈癌、乳腺癌检查。(8)为困难群体和农民工办理法律援助案件1200件、法律援助事项12000件；为城区80岁以上高龄老人按月发放生活津贴。(9)新增农民健身工程100个、社区健身路径工程100个、登山健身步道3条。(10)创建50所义务教育阶段标准化学校；按幼儿在园人数5%的比例给予困难家庭的入园幼儿每年1000元的资助。

与此同时，启动十三五规划前期研究工作。积极支持驻黄部队建设，促进军民融合发展。继续做好统计、编制、妇女儿童、青年、史志、气象、防震减灾、外事、侨务、对台、保密、档案、民族、宗教、科普和对外援助等工作。

各位代表!全面完成2014年经济社会发展各项目标任务，必须大力加强政府自身建设，努力建设法治政府和服务型政府。扎实开展党的群众路线教育实践活动，认真落实《党政机关厉行节约反对浪费条例》等中央、省市委有关作风建设规定，着力整治形式主义、官僚主义、享乐主义和奢靡之风，切实改进工作作风。出台《加强政府执行力的意见》，完善工作落实责任体系，创新重点工作专题推进和督办机制，加大问责力度，坚决查处有令不行、有禁不止行为，切实提高政府执行力。加快法治政府建设进程，制订实施《黄石市法治政府建设五年规划》，健全行政决策机制和程序，推进决策科学化、民主化、法治化；自觉接受市人大及其常委会的法律监督、工作监督和市政协的民主监督，切实做好人大议案、建议和政协提案办理工作。抓好党风廉政建设，严格执行党风廉政建设各项规定，强化对重要领域、关键岗位的行政监察和审计监督，坚决查处各类违纪违法案件，以反腐倡廉的实际成效取信于民。

各位代表!黄石已踏上全面深化改革、转型跨越发展的伟大征程!任何困难都改变不了黄石人民创造幸福生活的美好愿望，任何挑战都阻挡不了黄石人民加快跨越赶超的前进步伐。让我们紧密团结在以习近平同志为总书记的党中央周围，在市委的正确领导下，真抓实干、埋头苦干，夙夜在公、勤勉工作，为黄石加快建成鄂东特大城市、全面建成小康社会而努力奋斗!
